# Supplementary figures and images for: 4C-ker: A Method to Reproducibly Identify Genome-Wide Interactions Captured by 4C-Seq Experiments
Source: PLoS Comput Biol. 2016 Mar 3;12(3):e1004780. doi: 10.1371/journal.pcbi.1004780 (PMC4777514; doi:10.1371/journal.pcbi.1004780)

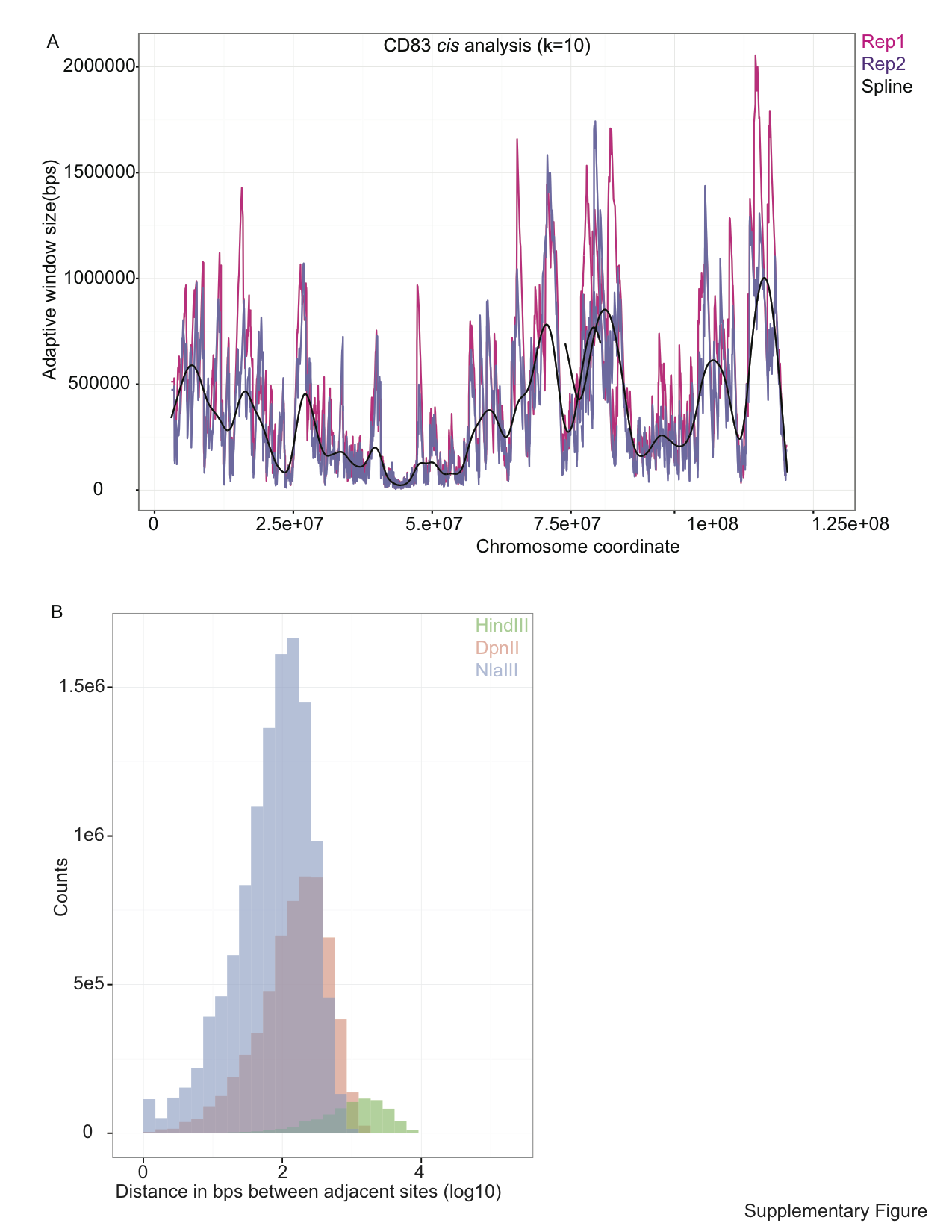

Supplement: S1 Fig — (A) Adaptive window analysis for the cis chromosome using k = 10. A spline is fitted to the two replicates (Cd83 dataset) to generate a smooth curve which is then used to create the overlapping windows. (B) Histogram of the distance between adjacent restriction enzyme sites in the mouse mm10 genome. NlaIII has the highest number of sites in the genome resulting in shorter distances between adjacent sites. (TIFF) [file pcbi.1004780.s001.tiff]

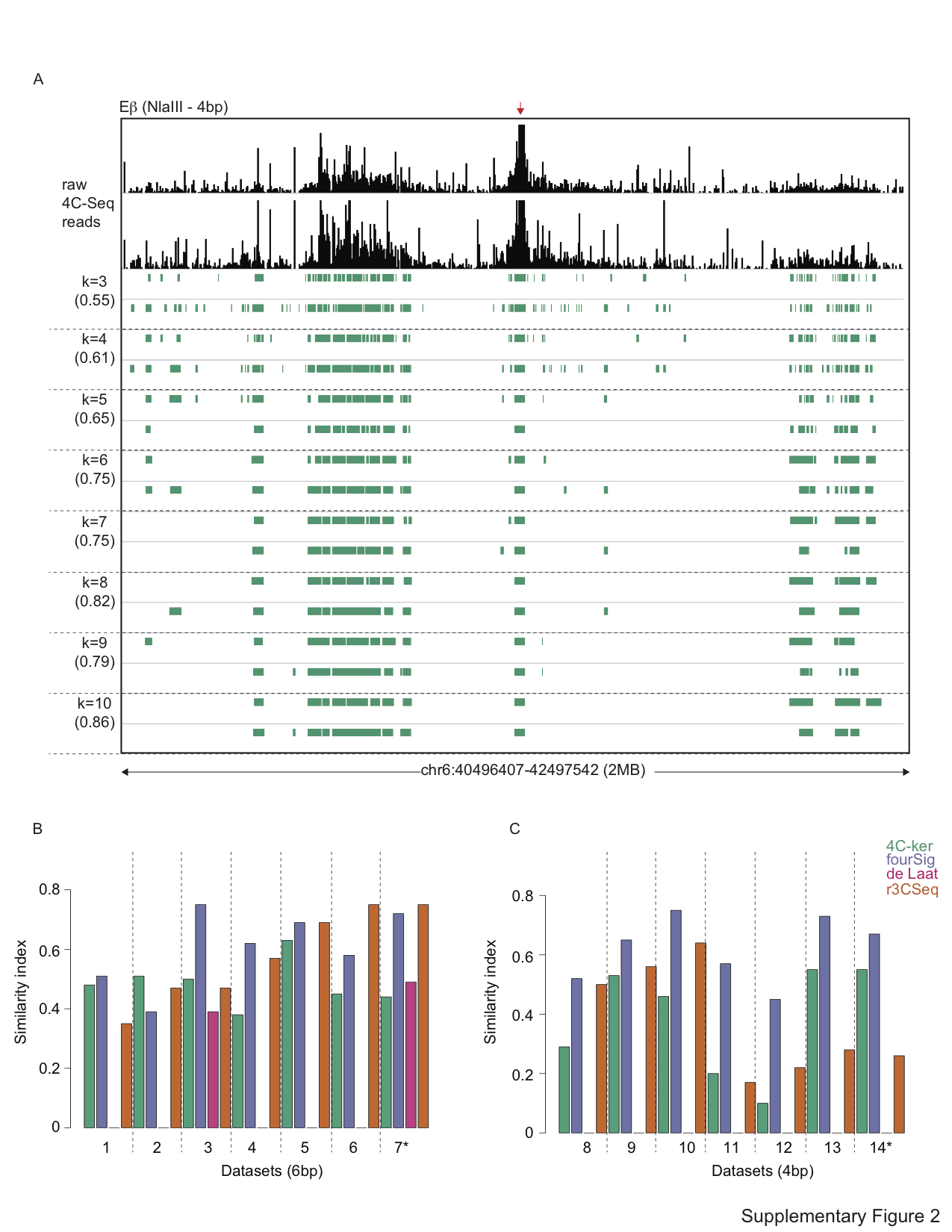

Supplement: S2 Fig — (A) Near bait analysis using different values of k for the 2MB region around the Eβ bait in DN cells. Domains called for the two replicates are shown and the similarity index below each value of k. (B-C) Similarity index between replicates for interacting domains identified in the region around the bait for 6bp and 4bp cutter datasets respectively. The numbers in the x-axis refer to each dataset. * Represents datasets shown in Fig 2A. (TIFF) [file pcbi.1004780.s002.tiff]

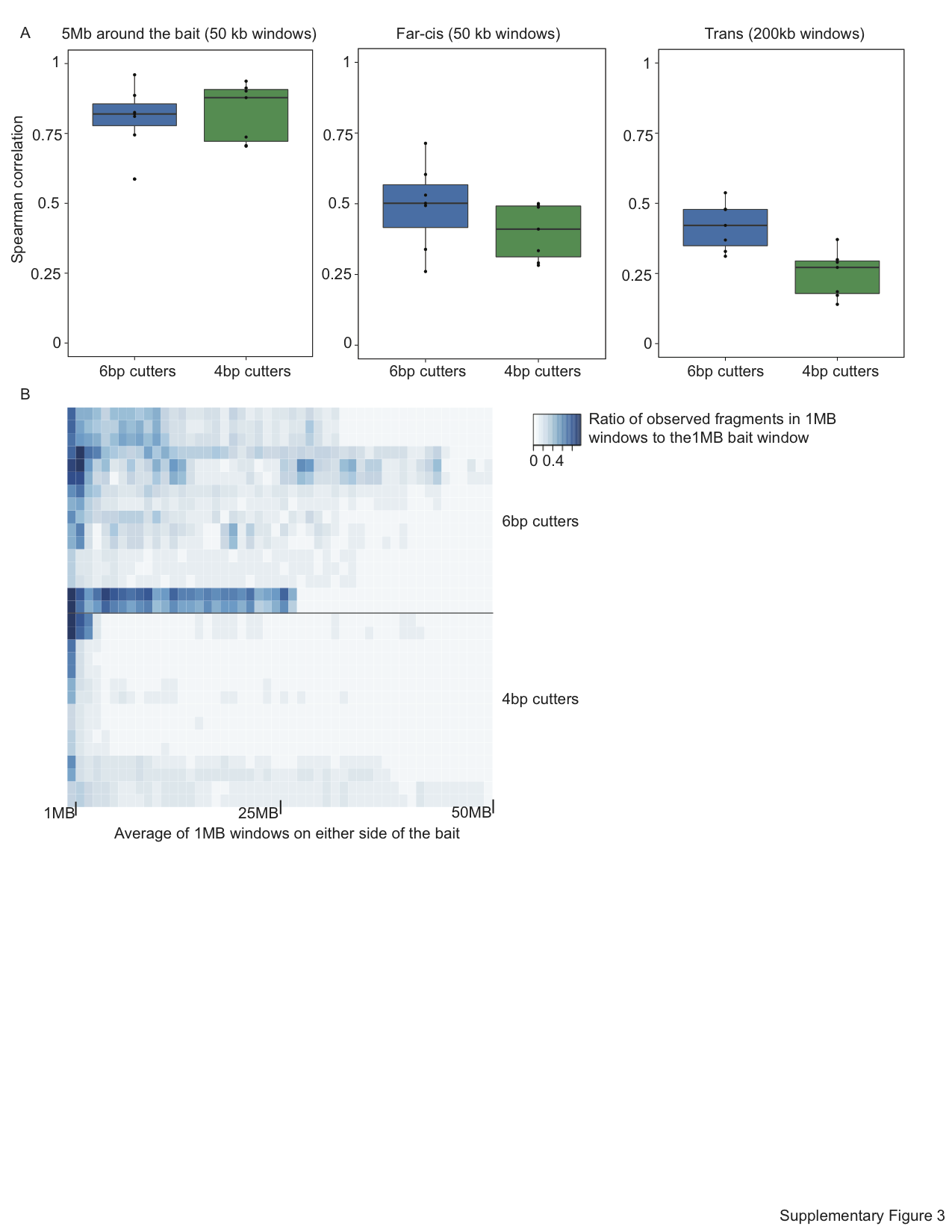

Supplement: S3 Fig — (A) Raw counts for different window sizes were used to calculate Spearman correlation across several datasets (listed in S1 Table). The mean of pairwise correlations were plotted for datasets with greater than 2 replicates. (B) Ratio of observed fragments in 1 MB windows (up to 50MB away from the bait) against the observed fragments in the 1MB window encompassing the bait. Each row represents a 4C-Seq experiment (replicates are separated). (TIFF) [file pcbi.1004780.s003.tiff]

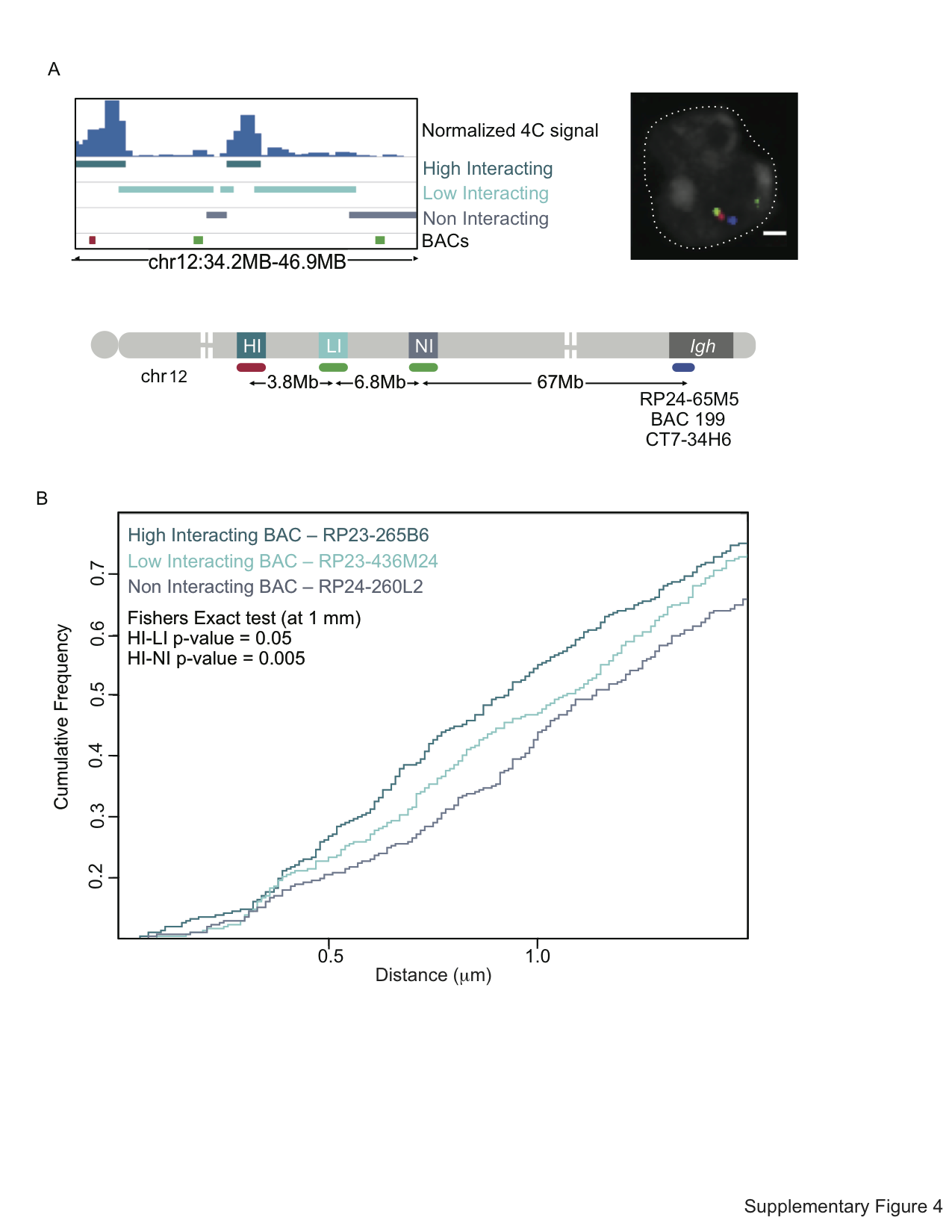

Supplement: S4 Fig — (A) Browser view of a far-cis region on chromosome 12 showing domains identified as High, Low and Non interacting states and the location of BACs chosen to label these regions as well as the distances separating them from each other and from Igh. These BACs, together with probes labeling the constant region of Igh were used for 3D-FISH on activated B cells. (B) The distance from each BACs to Igh was measured and plotted as a cumulative frequency curve. A shift to the left represents closer proximity to Igh. The BAC representing the High interacting state is more frequently found closer to Igh than the BACs representing the Low and Non interacting states. This difference is statistically significant using a Fisher’s exact test at 1μm distance. The FISH example shows one Z plane where one chromosome 12 is visible. (TIFF) [file pcbi.1004780.s004.tiff]

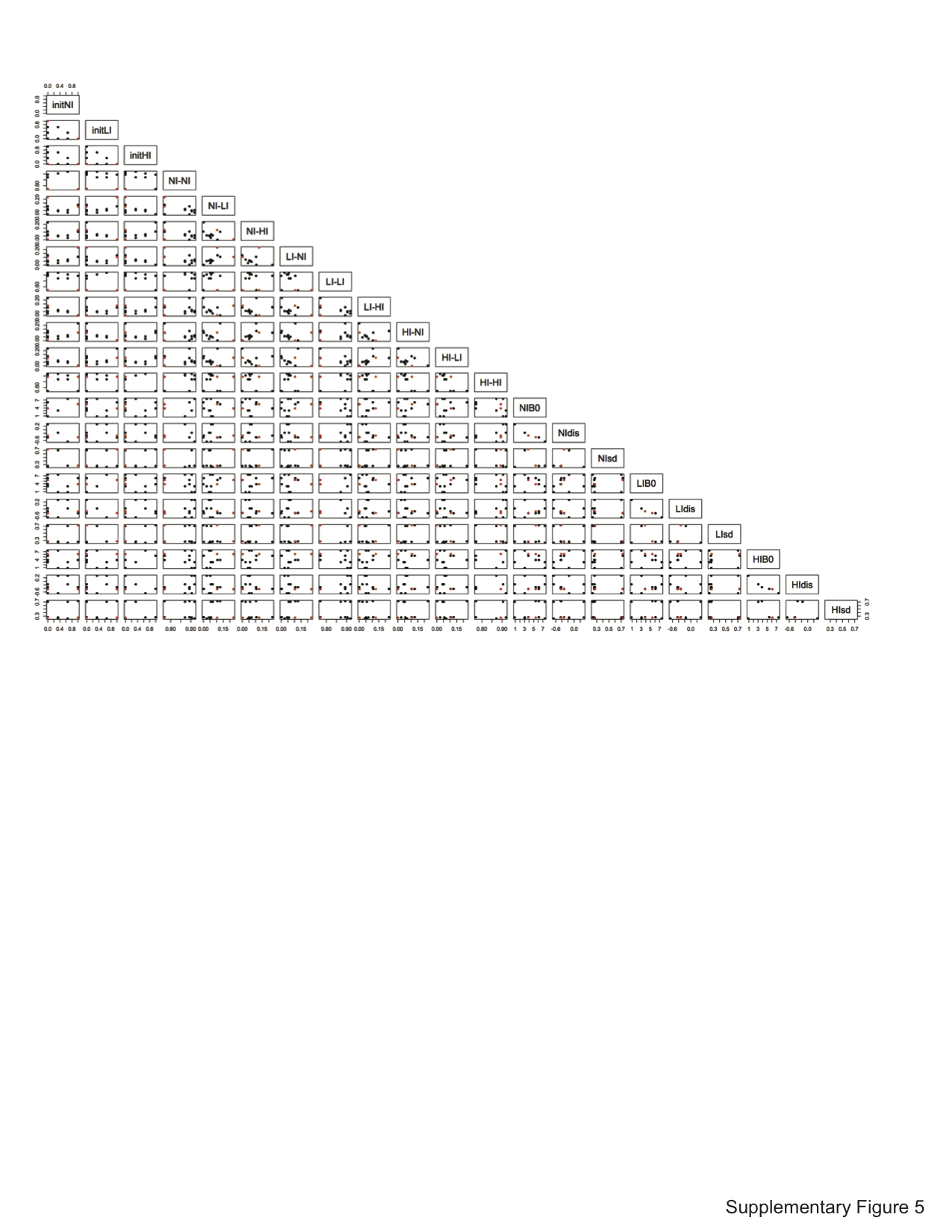

Supplement: S5 Fig — Estimation was performed using the EM algorithm with no constraints. The set of parameters that resulted in Viterbi calls with a reproducibility of 60% or greater across replicates are colored in red. The probability of transitioning to the same state is always higher than transitioning to a different state. As expected, the distance covariate term (names here as dis) is always negative for the reproducible set of parameters, confirming the decrease in signal with increase linear distance from the bait. (TIFF) [file pcbi.1004780.s005.tiff]

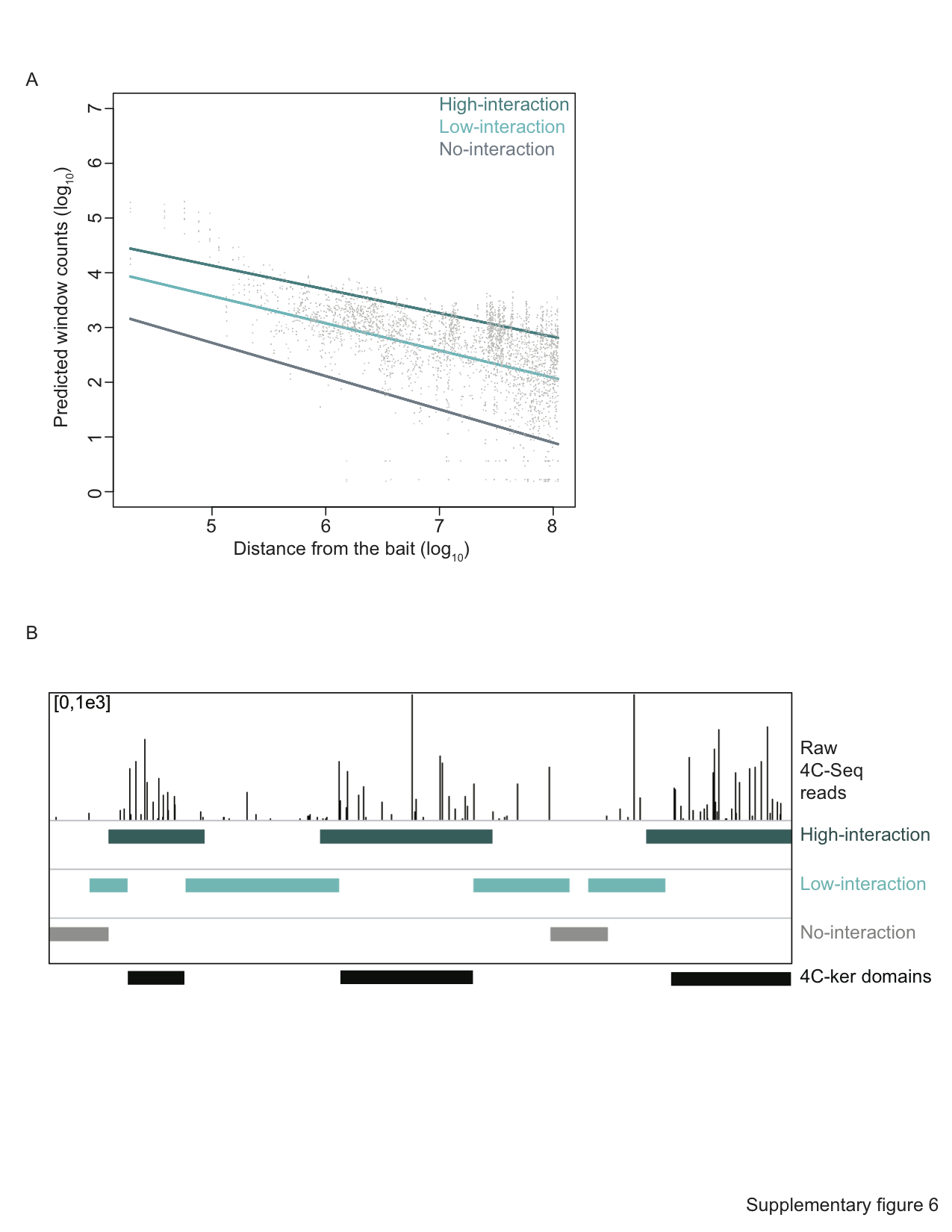

Supplement: S6 Fig — (A) Using the distance from the bait, the window counts were predicted from the estimated linear model for each of the HMM states. (B) Region of the bait chromosome showing the hidden states inferred by the Viterbi algorithm and the trimmed 4C-ker domains. (TIFF) [file pcbi.1004780.s006.tiff]

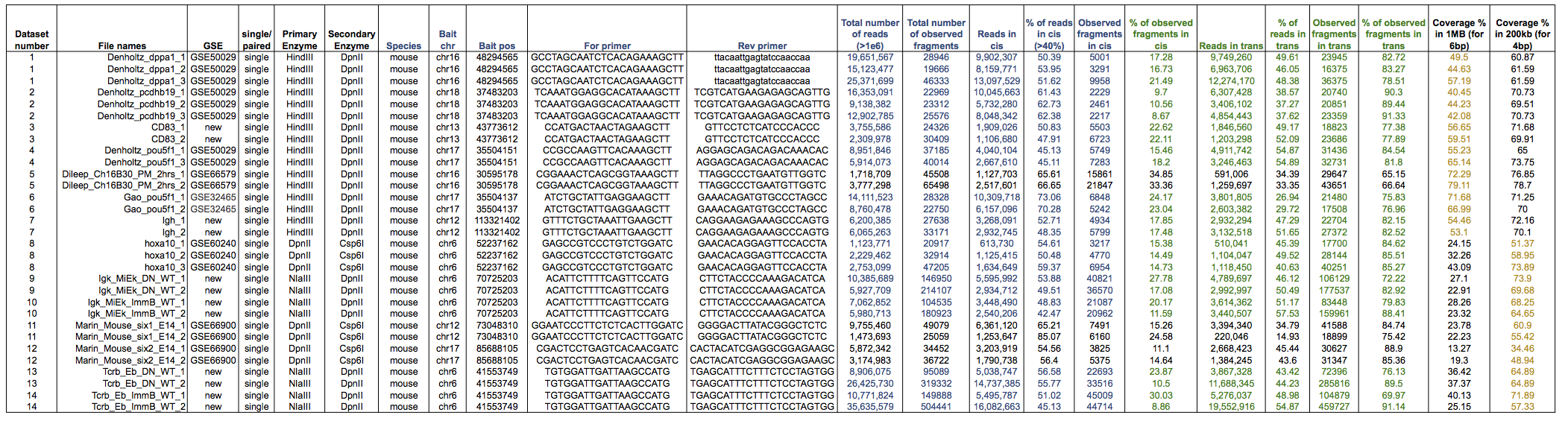

Supplement: S1 Table — (TIF) [file pcbi.1004780.s007.tif]
